# Study on the effectiveness of sulfate-reducing bacteria to remove Pb(II) and Zn(II) in tailings and acid mine drainage

**DOI:** 10.3389/fmicb.2024.1352430

**Published:** 2024-03-28

**Authors:** Yanrong Dong, Ziqing Gao, Junzhen Di, Dong Wang, Zhenhua Yang, Xuying Guo, Xiaotong Zhu

**Affiliations:** ^1^College of Civil Engineering, Liaoning Technical University, Fuxin, China; ^2^School of Mining, Liaoning Technical University, Fuxin, China; ^3^College of Science, Liaoning Technical University, Fuxin, China

**Keywords:** sulfate-reducing bacteria (SRB), treatment, tailing sands, heavy metals, acid mine

## Abstract

In view of water and soil getting polluted by Pb(II), Zn(II), and other heavy metals in tailings and acid mine drainage (AMD), we explored the removal effect of sulfate-reducing bacteria (SRB) on Pb(II), Zn(II), and other pollutants in solution and tailings based on the microbial treatment technology. We used the scanning electron microscope-energy dispersive spectroscopy (SEM-EDS), X-ray diffraction (XRD), and X-ray fluorescence (XRF), to reveal the mechanism of SRB treatment of tailings. The results showed that SRB had a strong removal capacity for Zn(II) at 0–40 mg/L; however, Zn(II) at 60–100 mg/L inhibited the growth of SRB. Similarly, SRB exhibited a very strong ability to remove Pb(II) from the solution. At a Pb(II) concentration of 10–50 mg/L, its removal percentage by SRB was 100%. SRB treatment could effectively immobilize the pollutants leached from the tailings. With an increase in the amount of tailings added to each layer, the ability of SRB to treat the pollutants diminished. When 1 cm of tailingssand was added to each layer, SRB had the best effect on tailing sand treatment. After treatment, the immobilization rates of ${\text{SO}}_{4}^{2-}$, Fe(III), Mn(II), Pb(II), Zn(II), Cu(II), and total Cr in the leachate of #1 tailing sand were 95.44%, 100%, 90.88%, 100%, 96.20%, 86.23%, and 93.34%, respectively. After the tailings were treated by SRB, although the tailings solidified into a cohesive mass from loose granular particles, their mechanical strength was <0.2 MPa. *Desulfovibrio* and *Desulfohalotomaculum* played the predominant roles in treating tailings by mixing SRB. The S^2−^ and carbonate produced by mixing SRB during the treatment of tailings could metabolize sulfate by combining with the heavy metal ions released by the tailings to form FeS, MnS, ZnS, CuS, PbS, Cr_2_S_3_, CaCO_3_, MnCO_3_, and other precipitated particles. These particles were attached to the surface of the tailings, reducing the environmental pollution of the tailings in the water and soil around the mining area.

## Graphical Abstract

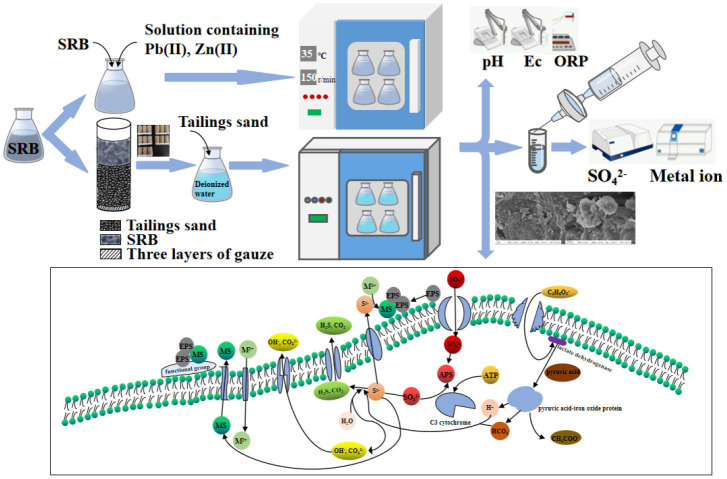

## Highlights

- SRB had a strong removal capacity for Zn(II) in the concentration range of 0–40 mg/L.
- SRB had a very strong ability to remove Pb(II).
- SRB treatment can effectively reduce environmental pollution.
- SRB can facilitate precipitation through metabolic activities.

## 1 Introduction

Mining activities cause immense acid mine drainage (AMD) containing ${\text{SO}}_{4}^{2-}$ and heavy metal ions (Nguyen et al., 2020). High concentrations of heavy metals and acidic pH in AMD pose major risks to surface water and groundwater, leading to the loss of biodiversity and deterioration of aquatic ecosystems (Rambabu et al., 2020). As AMD spreads further, it pollutes the surrounding water and soil and has a serious impact on the environment (Ryu et al., 2019; Watanabe et al., 2020). Consequently, heavy metal (Pb, Cd, Zn, etc.) pollution of soil and water has become a threat to human health in recent years (Kim et al., 2018; Zeng et al., 2022). For instance, according to China's soil pollution survey, 7% of agricultural soil is contaminated by Pb, which exceeds China's soil environmental quality standards (Yang et al., 2021). Pb, Zn, and other heavy metals can enter the food chain through rice, alfalfa, and other plants, causing serious harm to human health (Fang et al., 2020). These toxic metal ions are non-degradable, and their continuous presence in the ecosystem significantly pollutes the surface and groundwater resources, thus causing a serious public health challenge. Therefore, the remediation of water and soil containing Pb, Zn, and other heavy metal ions has become a research hot spot in recent years.

Bioremediation is an innovative technology for treating acidic heavy metal environmental pollution (Ayangbenro et al., 2018). Compared to traditional physical and chemical methods, bioremediation is an attractive alternative method (Razia et al., 2023). It is low-cost, maintains ecological balance, and helps rebuild the polluted environment (Martins et al., 2011). In particular, the sustainable bioremediation technology based on sulfate-reducing bacteria (SRB) is considered to be one of the best treatment schemes to alleviate environmental pollution caused by AMD (Alexandrino et al., 2011). Dissimilatory SRB is the main driving force behind AMD bioremediation (Chai et al., 2023). SRB can promote the conversion of sulfate into sulfide, which reacts with heavy metals to generate toxic metals and form a large number of metal sulfides. These metal sulfides are very stable and can be easily removed (Ayangbenro et al., 2018). The traditional chemical treatment of heavy metals often forms unstable metal hydroxide precipitation, making it difficult to recover these metals and causing secondary sludge disposal problems (Kumar et al., 2021). On the other hand, SRB can remove heavy metals and form insoluble salts (the Ksp of Zn, Cd, and Pb sulfides are 23.8, 26.1, and 28.0, respectively) even at very low pH (Su et al., 2022). This process promotes the application of SRB in the treatment of toxic heavy metal pollutants.

Nguyen et al. (2020) isolated an acid-resistant sulfate-reducing bacteria S_4_ from the mud of an AMD storage tank in Vietnam, which demonstrated great potential for remediating sulfate and heavy metals (Fe, Zn, Cu) in solution. Alexandrino et al. (2011) isolated a strain of SRB from a fumarole in Iceland, which showed very high sulfate reduction capacity in a mixture solution comprising 0.75 g/L iron, 0.20 g/L zinc, and 0.080 g/L copper. Lin et al. (2023) showed that the alkaline pretreatment of peanut shells as the SRB carbon source had a good removal effect of ${\text{SO}}_{4}^{2-}$ in solution and ${\text{SO}}_{4}^{2-}$ biological reduction load (140.61 mg/g). Gu et al. (2020) demonstrated that SRB could effectively remove Pd (II), Cd (II), and Ca (II) in solution, and the sediments (PbS, CdS, etc.) were very stable in wastewater. Nogueira et al. (2021) showed that SRB with bagasse as an electron donor could effectively remove ${\text{SO}}_{4}^{2-}$ and heavy metals in AMD, with removal rates of 55–91% for ${\text{SO}}_{4}^{2-}$ and 80%, 73%, and 60% for Zn, Cu, and Mn, respectively. Kumar and Pakshirajan (2021) reported that SRB could remove and recover heavy metals from synthetic wastewater containing Cd(II), Cu(II), Fe(III), Ni(II), Pb(II), and Zn(II). They had previously reported that when the influent concentration of Cu and Zn was 50 mg/L and 10 mg/L, respectively, they could be removed by SRB by more than 90% (Kumar and Pakshirajan, 2020). Hwang and Jho (2018) showed that native SRB isolated from soil samples in a mining area had a removal effect on the heavy metals and sulfates in the synthetic AMD prepared in the laboratory.

These studies establish that both SRB cultivated in the soil and pure SRB strains that were purchased had good removal effects on ${\text{SO}}_{4}^{2-}$ and heavy metal ions in simulated AMD solution. However, most of the ${\text{SO}}_{4}^{2-}$ and heavy metal ions in AMD come from the oxidation and dissolution release of minerals in the mine tailings. The acidic environment of AMD further promotes the dissolution of pollutants in tailings. The pollutants released from tailings are relatively complex, and laboratory simulations of AMD cannot fully reflect the remediation effect of SRB on pollutants. Especially, the fixation effect of SRB on pollutants in tailing sand is not clear. This limits the wide application of SRB in the field of environmental remediation in mining areas.

Therefore, this paper used soil to enrich SRB and discuss the immobilization effect SRB had on Pb(II), Zn (II), ${\text{SO}}_{4}^{2-}$, and other pollutants in a simulated AMD solution containing Pb(II) and Zn(II) and tailings. Using SEM-EDS, XRF, XRD, and other tests, the mechanism of SRB fixing pollutants in tailings was revealed. The results provide the technical reference for SRB effectively and sustainably repair AMD and tailings.

## 2 Materials and methods

### 2.1 Test materials

#### 2.1.1 Chemical reagent

The chemical reagents used in the experiments were all purchased from Tianjin Zhiyuan Chemical Reagent Co., Ltd. Deionized water prepared using the YL-400BU ultrapure water system was used to prepare the required solutions.

The composition of 1 L of modified Starkey medium included the following: 0.5 g K_2_HPO_4_, 1.0 g NH_4_Cl, 2.0 g MgSO_4_·7H_2_O, 0.5 g Na_2_SO_4_, 0.1 g CaCl_2_·H_2_O, 1.0 g yeast extract, 4 mL sodium lactate, 0.5 g (NH_4_)_2_Fe(SO_4_)_2_·6H_2_O, and 0.1 g (Dong et al., 2020) ascorbic acid. The pH of the medium was adjusted to 7.0. Except for (NH_4_)_2_Fe(SO_4_)_2_·6H_2_O and ascorbic acid, other chemicals were dissolved in deionized water and sterilized at 121°C for 30 min. Since (NH_4_)_2_Fe(SO_4_)_2_·6H_2_O and ascorbic acid cannot be sterilized at high temperatures, they were filtered through a 0.22 μm filter membrane for sterilization.

The solution containing Zn(II) was prepared with ZnSO_4_·7H_2_O, and its pH value was adjusted to 5 using 1 mol/L of HNO_3_ and 1 mol/L of NaOH. The solution containing Pb(II) was prepared with Pb(NO_3_)_2_.

#### 2.1.2 Bacterial culture

The SRB used in the experiment was cultivated from wet mud from Fuxin City, Liaoning Province (121° 41′ E, 41° 59′ N) (Dong et al., 2020). SRB in the logarithmic growth phase was used for the batch experiment. 5 g of seed sludge was added to 120 mL of sterilized modified Starkey medium. The solution was sealed with sterile liquid paraffin and a rubber stopper to create an anaerobic environment. The above solutions were cultured in a constant temperature oscillation incubator (HZ-9811K, Changzhou Langyue Instrument Manufacturing Co., Ltd., Jiangsu, China) at 35°C and 150 r/min. When the medium turned black, and the smell of rotten eggs emanated when the rubber plug was opened, it indicated that the mixed SRB had been cultured (Dong et al., 2020). The mixed SRB in the logarithmic growth phase was selected for the batch experiments.

**Figure 1:**
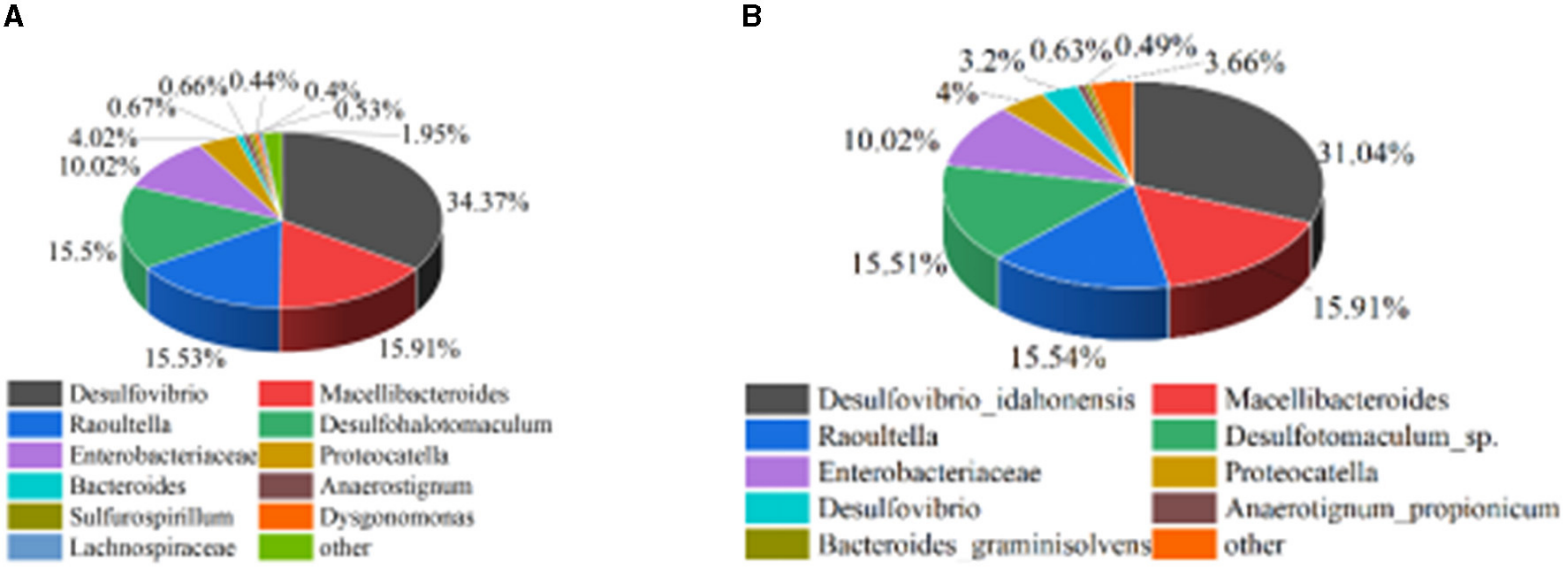
Microbial community structure analysis of mixed SRB. **(A)** Genus level. **(B)** Species level.

The enriched mixed SRB solution was sent to Emer Biotechnology (Xiamen) Co., Ltd. for microbial community structure analysis. The results are shown in Figure 1. It can be seen from Figure 1 that both *Desulfovibrio* and *Desulfotomaculum* were at the genus level of SRB and can metabolize sulfate. Among them, *Desulfovibrio* belonged to δ*-proteobacteria*, and *Desulfotomaculum* belonged to *Clostridium*. The relative abundance of *Desulfovibrio_idahonensis* in *Desulfovibrio* was 31.04%, and the relative abundance of *Desulfotomaculum_sp*. in *Desulfohalotomaculum* was 15.51%. It showed that *Desulfovibrio* and *Desulfotomaculum* were the dominant bacteria in the enriched mixed SRB.

### 2.2 Test materials

#### 2.2.1 Batch experiments

A series of batch tests were used to explore the removal of Zn(II) and Pb(II) by SRB. The test steps for removing Zn(II) by SRB included inoculating SRB into the solution with pH=5 and Zn(II) concentrations of 20 mg/L, 40 mg/L, 60 mg/L, 80 mg/L, and 100 mg/L. The volume ratio of SRB to the solution containing Zn(II) was 1:20. The solutions were sealed with sterile liquid paraffin and a rubber stopper. After placing the conical flasks in a constant temperature oscillation incubator (HZ-9811K type) at 35°C and 150 r/min, samples were taken for testing after incubation for a certain time. The sterile medium was used as the blank group. Three replicates were made for each test sample. The sample was filtered with a 0.22 μm filter membrane, and the filtered sample was tested for ${\text{SO}}_{4}^{2-}$ and metal ion concentration. Finally, the percentage removal rate of ${\text{SO}}_{4}^{2-}$ and Zn(II) was calculated. The test procedure for removing Pb(II) by SRB was similar to the above-mentioned process. Pb(II) was prepared with Pb(NO_3_)_2_, and the initial concentration of Pb(II) was 10 mg/L, 20 mg/L, 30 mg/L, 40 mg/L, and 50 mg/L.

#### 2.2.2 Experiment of SRB treatment on tailing sand

**Figure 2:**
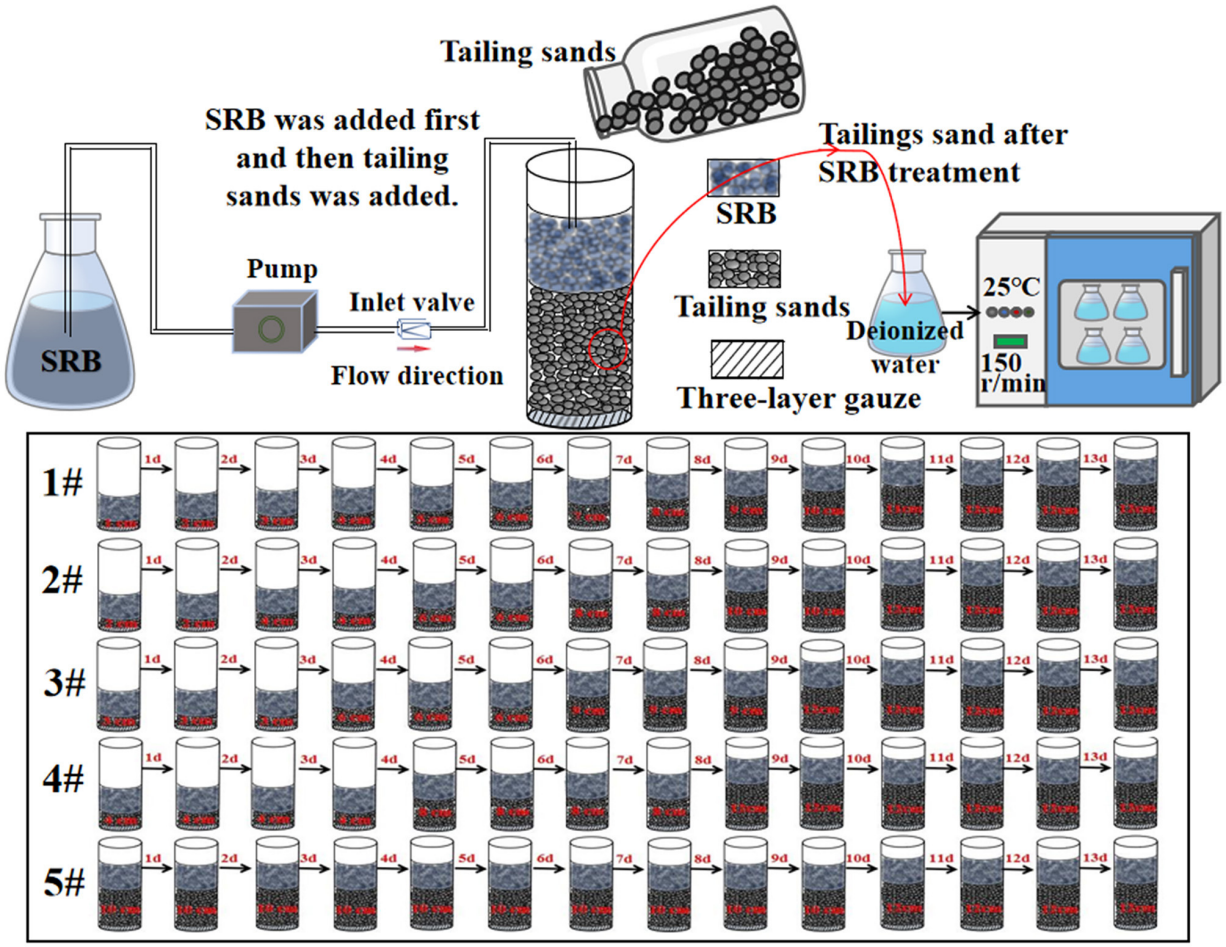
Schematic diagram of the experimental setup for SRB-treated tailings.

Test method for SRB to treat tailing sand: tailing sand was taken from a lead-zinc mine enterprise in Huludao City, Liaoning Province, China (120°38′E, 40°56′N). 60–100 mesh size tailing sand was screened for testing. An acrylic tube with an inner diameter of 50 mm and height of 170 mm was used as the mold in the test. The bottom end of the mold was sealed with three layers of gauze. Every day, 1 cm of tailing sand was added into the #1 mold. In the #2 mold, 2 cm of tailing sand was added every 2 days. Similarly, 3 cm of tailing sand was into the #3 mold every 3 days and 4 cm into the #4 mold every 4 days. In the #5 mold, 10 cm of tailing sand was added once on the first day and then 2 cm on day 11. When the tailing sand in molds #1-5 reached a height of 12 cm, we stopped adding any more. 150 mL of SRB was injected into all five molds every day for 13 consecutive days, and three repetitions were made for each group. The schematic diagram of the experimental setup for SRB treatment of tailing sand is shown in Figure 2. Prior to drying, samples of treated #1 tailing sand (the wet tailing sand at the bottom of the cylinder) were sent to Emerson Biotech (Xiamen) Co., Ltd. for microbial community structure analysis. The tailing sand samples treated with SRB in the 15 cylinders were removed from the molds. They were forced dried in an air drying oven (model GZX-9246MBE) at 105°C. The tailing sand cylinders were then ground with sandpaper to produce specimens 5 cm in diameter and 10 cm in height. These specimens were placed on a TH-8100 universal testing machine and subjected to unconfined compressive strength (UCS) tests at a loading rate of 2 mm/min. After UCS testing, the tailing sand were ground and sieved, and the 60–100 mesh fraction of SRB-treated tailing sand was subjected to leaching tests for pollutant analysis. The tailings in molds #1-5 were respectively recorded as #1-5. They were then added to deionized water according to the solid-liquid ratio of 1 g:5 mL and subject to leaching test at 25°C and 150 r/min. Tailings without SRB treatment were used as the blank group in the test. The leachate was measured every day for its pH value and the concentration of iron, manganese, copper, zinc, lead, cadmium, chromium, and ${\text{SO}}_{4}^{2-}$. The tailing sand from mold #1 was selected as the experimental group, while tailing sand was used as the blank control group for SEM, EDS, and XRD testing.

#### 2.2.3 Analytical methods

Based on the electrode method (HJ 1147-2020), a PHS-3C pH meter was used to measure pH values. The oxidation-reduction potential (ORP) was measured with a CT-8022 ORP meter. Electrical conductivity (Ec) was measured with a pen-type Ec meter. The OD_600_ value was measured with a V-1600PC visible spectrophotometer. Based on the barium chromate spectrophotometry (HJ/T 342-2007), the concentration of ${\text{SO}}_{4}^{2-}$ was determined by a V-1600PC visible spectrophotometer. Based on the atomic absorption spectrophotometry (GB 7475-87), the concentrations of Cu, Zn, Pb, and Cd were determined by a Z-2000 flame atomic spectrophotometer. The elements of Fe and Mn were determined by flame atomic absorption spectrophotometry (GB 11911-89). Cr was determined by flame atomic absorption spectrophotometry (HJ 757-2015).

Zeiss Sigma 500 scanning electron microscope was used to detect the tailings by SEM, and EDS scanning energy spectrometer was used to detect the chemical substances on the surface of the samples. The tailing sand samples were ground to 200 mesh, and XRD detection was conducted. The scanning step for XRD detection with Bruker D8 Advance X-ray diffractometer was 5–90°.

## 3 Results and discussion

### 3.1 Effect of SRB on zn(II) removal

**Figure 3:**
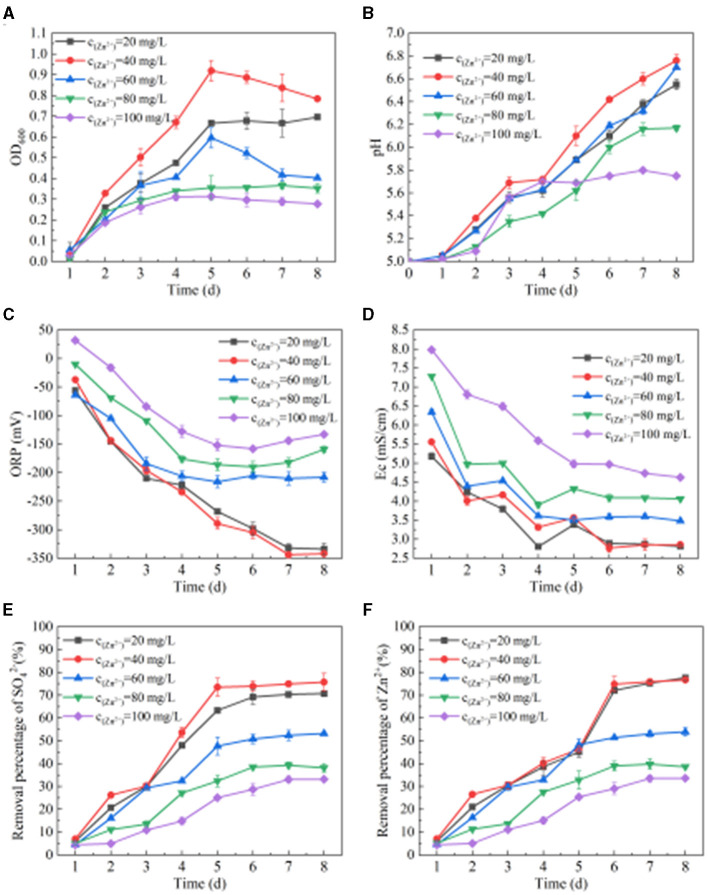
Effect of SRB on removing Zn(II). **(A)** Change of OD_600_ value. **(B)** Change of pH value. **(C)** Change of ORP value. **(D)** Change of Ec value. **(E)** ${\text{SO}}_{4}^{2-}$ removal percentage. **(F)** Zn(II) removed by SRB.

From Figure 3A, we can see the change in OD_600_ when SRB metabolizes Zn(II). When the concentration of Zn(II) is 20–60 mg/L, the OD_600_ value first increases and then decreases. When the concentration of Zn(II) is 80–100 mg/L, the OD_600_ value first increases and then stabilizes. When the concentration of Zn(II) is 20 mg/L, 40 mg/L, 60 mg/L, 80 mg/L, and 100 mg/L, the OD_600_ values are 0.67, 0.92, 0.60, 0.36, and 0.31, respectively, after adding SRB for 5 days. When the concentration of Zn(II) is 20–40 mg/L, the OD_600_ value gradually increases with the increase of Zn(II) concentration, indicating that proper increase of Zn(II) concentration within this range is conducive to stimulating SRB reproduction. When the initial concentration of Zn(II) is 40 mg/L and is increased to 100 mg/L, the OD_600_ value gradually decreases with the increase of Zn(II) concentration, indicating that Zn(II) in this concentration range inhibits the growth of SRB.

It can be seen from Figures 3B–D that when the concentration of Zn(II) is 20 mg/L, 40 mg/L, 60 mg/L, 80 mg/L, and 100 mg/L, respectively, the pH, ORP, and Ec values change after SRB is added to the solution containing Zn(II). After adding SRB for 8 days, the pH values are 6.55, 6.76, 6.70, 6.17, and 5.75, respectively. The ORP values are −334 mV, −342 mV, −208 mV, −159 mV, and −133 mV, respectively. The Ec values are 2.82 mS/cm, 3.46 mS/cm, 3.48 mS/cm, 4.06 mS/cm, and 4.43 mS/cm, respectively. With the extension of time, the ORP and Ec values generally show a downward trend. When the initial concentration of Zn(II) is 10–80 mg/L, the pH value generally shows an upward trend. When the concentration of Zn(II) is 80 mg/L and 100 mg/L, the rate of change in pH value is significantly reduced. In particular, when the concentration of Zn(II) is 100 mg/L, the pH value first increases slowly and then finally stabilizes at 5.7–5.8. When the initial concentration of Zn(II) is 80 mg/L and 100 mg/L, the rate of change in ORP and Ec values decreases significantly, indicating that a high concentration of Zn(II) inhibits SRB metabolism.

It can be seen from Figures 3E, F that when SRB metabolizes Zn(II), the removal percentage of ${\text{SO}}_{4}^{2-}$ and Zn(II) by SRB generally increases first and then tends to be stable. At 1–6 days, SRB metabolizes vigorously and reduces more ${\text{SO}}_{4}^{2-}$, and the removal percentage of ${\text{SO}}_{4}^{2-}$ and Zn(II) increases rapidly. At 6–8 days, SRB gradually enters the decay stage, and the removal percentage of ${\text{SO}}_{4}^{2-}$ and Zn(II) tends to be stable. Properly increasing the concentration of Zn(II) in the range of 0–40 mg/L will increase the activity of SRB, while Zn(II) in the range of 40-100 mg/L will inhibit the reduction of ${\text{SO}}_{4}^{2-}$ by SRB. When the initial concentration of Zn(II) is 20 mg/L, 40 mg/L, 60 mg/L, 80 mg/L, and 100 mg/L, the removal percentages of ${\text{SO}}_{4}^{2-}$ and Zn(II) are 70.71%, 75.72%, 53.21%, 38.21%, and 33.21%, and 77.61%, 76.69%, 53.89%, 38.70%, and 33.64%, respectively, after adding SRB for 8 days. It is reported that Zn(II) can precipitate in a weak acidic environment in the reactor for SRB removing heavy metals (Hedrich and Johnson, 2014). According to the change in pH values, pH value was not the main factor limiting the removal of Zn(II) by SRB. At the initial stage, Zn(II) reacts with S^2−^ produced by SRB metabolism to form ZnS, achieving the aim of removing Zn(II). With the reaction proceeding, the alkalinity produced by SRB metabolism also promoted the transformation from ZnS to ZnCO_3_ precipitation (Dvorak et al., 1992). Jong and Parry (2003) and others used silica sand as the filler and treated light heavy metal polluted water with mixed SRB strain for 14 days, and the removal percentage of Zn(II) at 5–50 mg/L reached 97.5%. In contrast, the removal percentage of SRB for 40 mg/L Zn(II) was significantly lower than the above removal percentage, mainly because the SRB in this study was floating in solution and had no attached carrier, and the bacterial activity was easily affected by sulfide and heavy metal ions (Bijmans et al., 2009; Barbosa et al., 2014). Wang (2014) reported that when the concentration of Zn(II) was >45 mg/L, it inhibits SRB. The results of this study were similar to those of the above studies.

To sum up, SRB has a strong Zn(II) removal capacity when it is in the concentration range of 0–40 mg/L. If the initial concentration of Zn(II) is 60–100 mg/L, then the growth of SRB would be inhibited. The tolerance concentration of SRB to Zn(II) was found to be 40 mg/L. At this concentration, the maximum values of OD_600_, pH value, ${\text{SO}}_{4}^{2-}$ fixation rate, and Zn(II) fixation rate were 0.92, 6.76, 75.72%, and 76.69% respectively, and the minimum values of ORP and Ec were −344 mV and 2.77 mS/cm respectively.

### 3.2 Effect of SRB on Pb(II) removal

**Figure 4:**
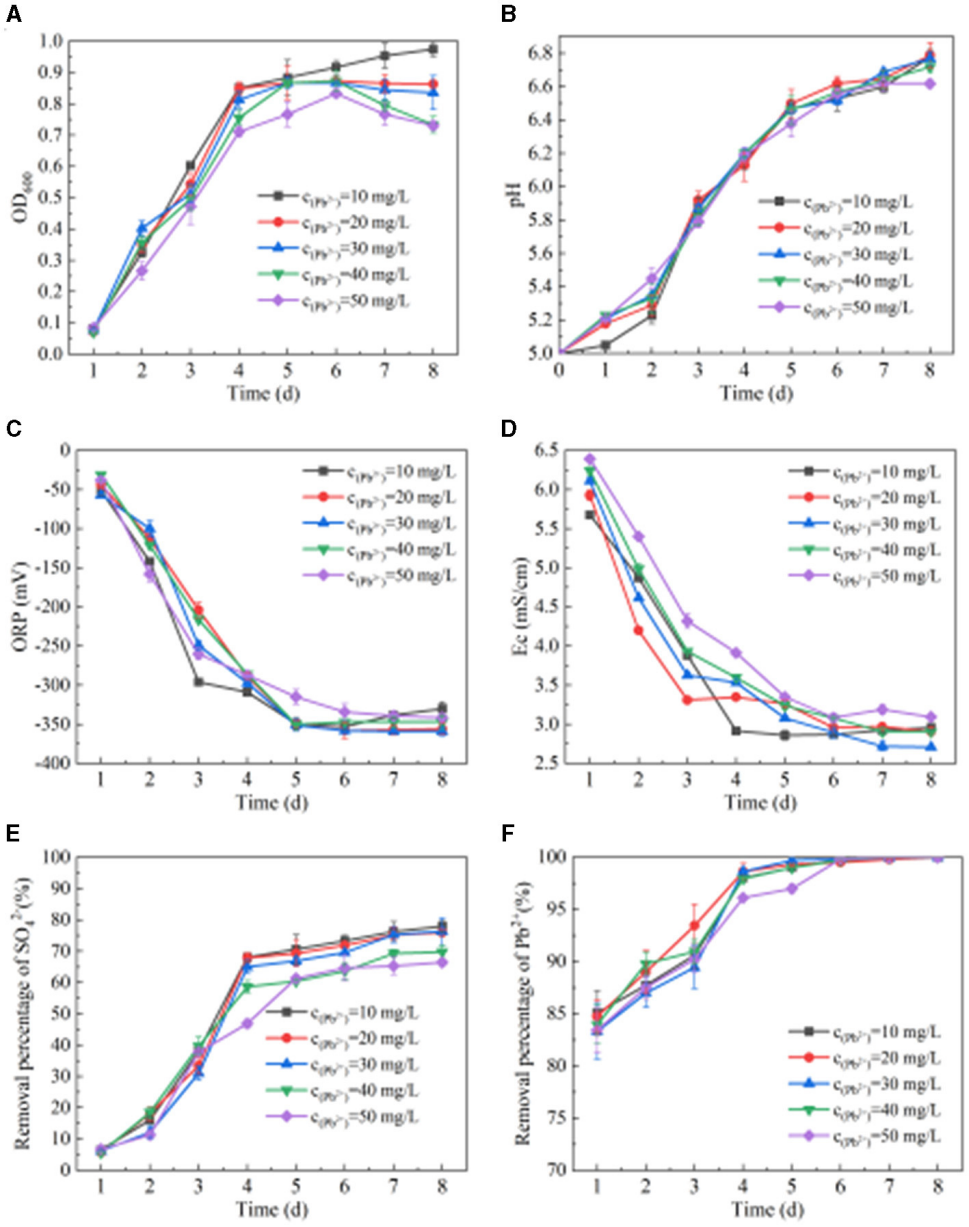
Effect of SRB on Pb(II) removal. **(A)** Change of OD_600_ value. **(B)** Change of pH value. **(C)** Change of ORP value. **(D)** Change of Ec value. **(E)** ${\text{SO}}_{4}^{2-}$ removal percentage. **(F)** Pb(II) removed by SRB.

From Figure 4A, we can see the change in OD_600_ when SRB metabolizes Pb(II). When the concentration of Pb(II) is 10 mg/L, the OD_600_ value shows an upward trend. When the concentration of Pb(II) is 20 mg/L, the OD_600_ value first increases and then stabilizes. When the concentration of Pb(II) is 30–50 mg/L, the OD_600_ value shows a trend of increasing first and then decreasing slightly. When the Pb(II) concentration is 10 mg/L, 20 mg/L, 30 mg/L, 40 mg/L, and 50 mg/L, respectively, the OD_600_ values are 0.92, 0.87, 0.87, 0.87, and 0.83 after SRB addition for 6 days. It can be seen from Figures 4B–D that when the concentration of Pb(II) is 10 mg/L, 20 mg/L, 30 mg/L, 40 mg/L, and 50 mg/L, respectively, the pH, ORP, and Ec values change after SRB is added to the solution containing Pb(II). After adding SRB for 8 days, the pH values are 6.78, 6.79, 6.77, 6.72, and 6.62, respectively. The ORP values are −330 mV, −356 mV, −359 mV, −347 mV, and −341 mV, respectively. The ORP values all dropped below −330 mV, while the Ec values were 2.96 mS/cm, 2.91 mS/cm, 2.71 mS/cm, 2.91 mS/cm, and 3.10 mS/cm, respectively. With the extension of time, the pH value generally shows an upward trend, while the ORP and Ec values generally show a downward trend. With the change of initial Pb(II) concentration, the changes in pH, ORP, and Ec values are similar, indicating that a change in Pb(II) concentration has little impact on SRB metabolism at this time. It can be seen from Figures 4E, F that when SRB metabolizes Pb(II), the removal percentage of ${\text{SO}}_{4}^{2-}$ and Pb(II) by SRB generally increases first and then tends to be stable. When the initial Pb(II) concentration is 10 mg/L, 20 mg/L, 30 mg/L, 40 mg/L, and 50 mg/L, respectively, the removal percentage of ${\text{SO}}_{4}^{2-}$ is 77.95%, 76.04%, 76.33%, 69.81%, and 66.45% after adding SRB for 8 days. The final removal percentage of Pb(II) reached 100%, indicating that SRB has a very strong ability to remove Pb(II).

### 3.3 Effect and mechanism of SRB treatment of tailings

**Figure 5:**
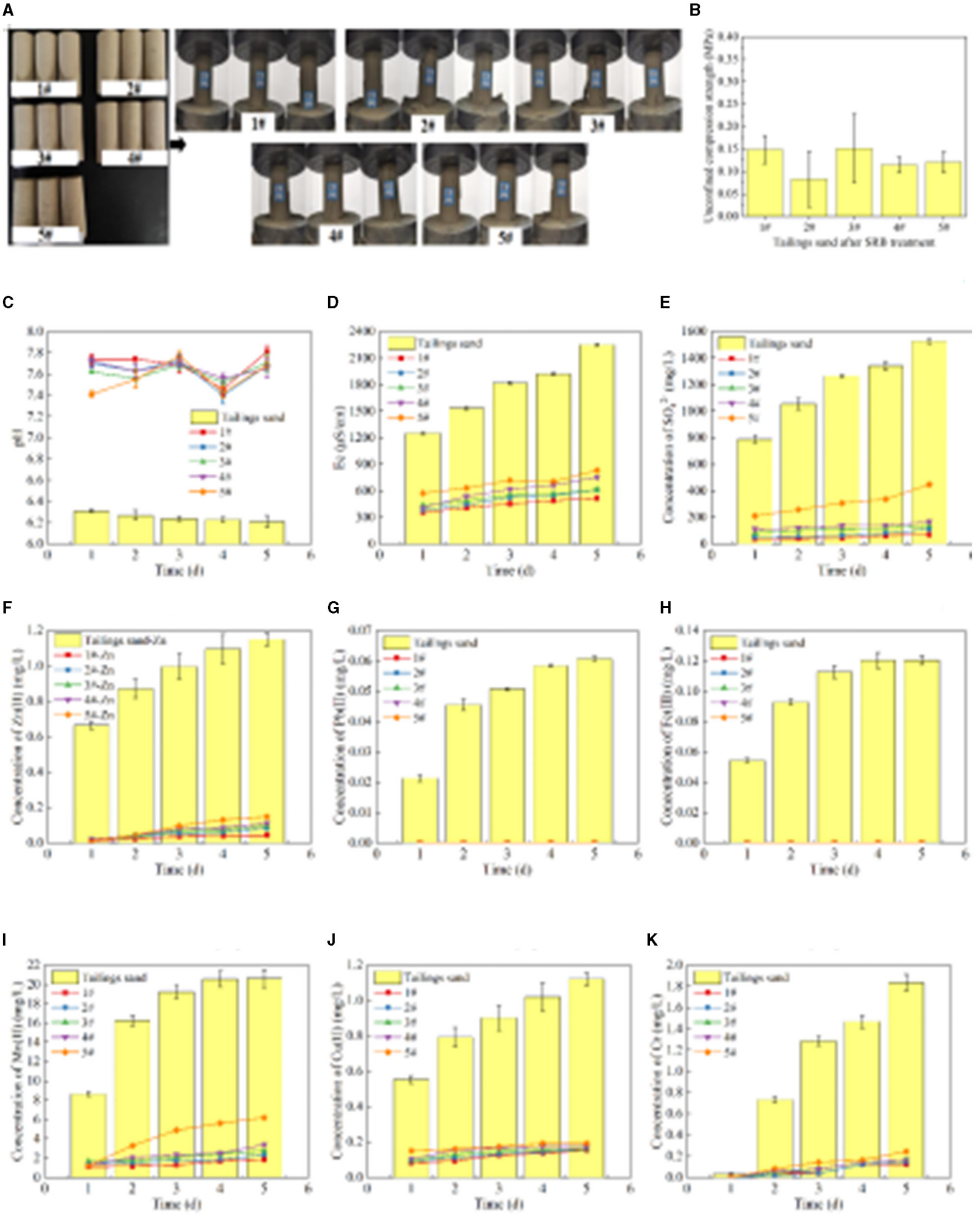
The UCS after SRB treatment of tailings and leaching of pollutants from tailing sand. **(A)** The specimen formed after SRB treatment of tailings sand and the UCS test diagram of the specimen. **(B)** UCS of the specimen. **(C)** Leachate pH value. **(D)** Leachate Ec value. **(E)** Leaching concentration of ${\text{SO}}_{4}^{2-}$. **(F)** Leaching concentration of Zn(II). **(G)** Leaching concentration of Pb(II). **(H)** Leaching concentration of Fe(III). **(I)** Leaching concentration of Mn(II). **(J)** Leaching concentration of Cu(II). **(K)** Leaching concentration of total chromium.

The specimens in molds #1-5 formed after treating the tailing sand with SRB are shown in Figure 5A, and the UCS results are shown in Figure 5B. It can be observed from Figures 5A, B that the tailing sand, after SRB treatment, transforms from a loose granular state to a cohesive whole. The UCS values of specimens #1-5 are 0.15 MPa, 0.08 MPa, 0.15 MPa, 0.12 MPa, and 0.12 MPa, respectively. Compared to the loose tailing sand, the UCS values are enhanced after SRB treatment, but the relatively small increase in UCS results in large errors in the three parallel tests. The UCS values of #1-5 are all <0.2 MPa, indicating that the effectiveness of SRB bio-cementation in solidifying tailing sand particles is unsatisfactory.

It can be seen from Figure 5C that the pH value of the leachate of #1-5 tailing sand is stable at 7.41–7.81, which is weakly alkaline. However, the pH value of tailing leachate is stable at 6.21–6.31, showing weak acidity. It can be seen from the comparison of pH values that the pH value of tailing leachate increases after the SRB treatment of tailings. The main reason for the increase in pH value after SRB treatment is that SRB produces ${\text{HCO}}_{3}^{-}$ and other metabolites during sulfate metabolism. After the pH value is raised, when the tailing sand is stacked in the outdoor environment, the pollutants, such as heavy metals dissolved by rain and other actions, are reduced. According to Figure 5D, the Ec value of #1-5 tailing sand leachate is 354–832 μS/cm, while the Ec value of tailings leachate is 1,252–2,254 μS/cm. Compared with the Ec value of tailings leachate, the Ec value of tailings leachate after SRB treatment is significantly lower, indicating that SRB treatment can effectively inhibit the dissolution of soluble substances in tailings. Therefore, SRB treatment can effectively reduce the diffusion of pollutants in tailing sand and reduce the impact of tailing sand accumulation on the environment.

It can be seen from Figure 5E that the concentration of ${\text{SO}}_{4}^{2-}$ in tailing sand leachate increases with the leaching time. The concentration of ${\text{SO}}_{4}^{2-}$ is 1,528 mg/L after leaching tailings for 5 days. After #1-5 tailings leaching for 5 days, the concentrations of ${\text{SO}}_{4}^{2-}$ are 70 mg/L, 111 mg/L, 139 mg/L, 167 mg/L, 449 mg/L. Compared with the concentration of ${\text{SO}}_{4}^{2-}$ in the tailings leachate, the concentration of ${\text{SO}}_{4}^{2-}$ in the leachate after SRB treatment of tailings decreased by 95.44%, 92.73%, 90.87%, 89.07%, and 70.62% at day 5, respectively, indicating that SRB can effectively fix the ${\text{SO}}_{4}^{2-}$ in tailings. Comparing the ${\text{SO}}_{4}^{2-}$ concentration in the leachate of #1-5 tailing sand, it can be seen that with the increase of each layer's thickness of tailing sand, the ability of SRB to fix ${\text{SO}}_{4}^{2-}$ gradually decreases. This is because when the amount of tailings addition is small (#1-4), SRB has sufficient contact with tailings, and SRB can convert ${\text{SO}}_{4}^{2-}$ released by dissolution of tailings into sulfide precipitation. However, when the amount of tailings addition is large (#5), the contact of SRB with the tailings at the bottom layer is limited, resulting in an increase in the overall ${\text{SO}}_{4}^{2-}$ leaching amount.

It can be seen from Figures 5F, G that the concentration of Zn(II) and Pb(II) in tailing leaching first increases and then tends to stabilize with the prolongation of leaching time. After 5 days of leaching, the concentrations of Zn(II) and Pb(II) in the leachate are 1.15 mg/L and 0.06 mg/L, respectively. However, after 5 days of leaching of #1-5 tailings, the concentration of Pb(II) in the leachate is 0 mg/L, and the concentration of Zn(II) is 0.044 mg/L, 0.083 mg/L, 0.097 mg/L, 0.11 mg/L, 0.15 mg/L respectively. Comparing the concentration of Pb(II) in the leachate, it can be seen that after SRB treatment of tailings, Pb(II) is completely fixed, and there is no secondary release of Pb(II). Compared with tailings, the concentration of Zn(II) in the leachate of #1-5 tailings after SRB treatment decreased by 96.20%, 92.76%, 91.58%, 90.34%, and 87.05%, respectively, on day 5, indicating that SRB can effectively repair the Zn(II) and Pb(II) pollution in tailings. Compared with #1-5, the ability of SRB to fix Zn(II) gradually decreases with the increase of the thickness of each layer of tailings.

It can be seen from Figures 5H, I that the concentration of Fe(III) and Mn(II) in tailings leaching firstly increases and then tends to stabilize with the leaching time. After 5 days of leaching, the concentrations of Fe(III) and Mn(II) in the leachate of tailing sand are 0.12 mg/L and 20.61 mg/L, respectively. However, after 5 days of leaching #1-5 tailing sand, the concentration of Fe(III) in the leachate is 0 mg/L, and the concentrations of Mn(II) are 1.88 mg/L, 2.35 mg/L, 2.76 mg/L, 3.40 mg/L and 6.20 mg/L respectively. By comparing the concentration of Fe(III) in the leachate, it can be seen that after SRB treatment of tailings, Fe(III) in the tailing sand is completely immobilized, and there is no dissolution released. Compared with tailings, the concentration of Mn(II) in the leachate of #1-5 tailings after SRB treatment decreased by 90.88%, 88.60%, 86.62%, 83.50%, and 69.91%, respectively, on day 5, indicating that the use of SRB can effectively repair Fe(III) and Mn(II) pollution in tailings. With the increase of the thickness of each layer of tailings, the ability of SRB to fix Mn(II) gradually decreases, which is related to the ability of SRB to fix ${\text{SO}}_{4}^{2-}$ in tailings. When SRB metabolizes more ${\text{SO}}_{4}^{2-}$, it produces more S^2−^ and ${\text{CO}}_{3}^{2-}$, and a large amount of S^2−^ and ${\text{CO}}_{3}^{2-}$ precipitates the Mn(II) in the solution. The fixation effect of SRB on Mn(II) in tailing sand is significantly lower than that of Fe(III), mainly due to the high concentration of Mn(II) released from tailing sand and the high solubility product of MnS and other precipitates.

It can be seen from Figures 5J, K that the concentration of Cu(II) and total Cr leached from tailing sand increases with the leaching time. After 5 days of leaching, the concentrations of Cu(II) and total Cr in the leachate of tailing sand are 1.12 mg/L and 1.84 mg/L, respectively. However, after #1-5 tailing sand leaching for 5 days, the concentrations of Cu(II) in the leachate are 0.15 mg/L, 0.16 mg/L, 0.16 mg/L, 0.18 mg/L and 0.19 mg/L respectively, and the concentrations of total Cr are 0.12 mg/L, 0.14 mg/L, 0.16 mg/L, 0.16 mg/L, and 0.24 mg/L, respectively. Compared with the tailings, the concentrations of Cu(II) in the leachate of #1-5 tailings after SRB treatment decreased by 86.23%, 85.74%, 85.74%, 84.27%, and 82.81% respectively on day 5, and the total Cr concentration decreased by 93.34%, 92.22%, 91.11%, 91.11%, and 86.67% respectively, indicating that SRB can effectively immobilize Cu(II) and total Cr in tailings. According to the comparison of #1-5, the ability of SRB to immobilize Cu(II) and total Cr gradually decreases with the increase of the thickness of each layer of tailings.

To sum up, compared with the tailings, the concentration of pollutants leached from #1-5 tailings after SRB treatment is significantly lower, indicating that SRB has a good fixation effect on soluble ${\text{SO}}_{4}^{2-}$, Fe(III), Mn(II), Pb(II), Zn(II), Cu(II), total Cr and other pollution ions in tailings. SRB can effectively reduce the impact of dissolved pollutants and the release of tailing sand to the environment. At the same time, when comparing the different concentrations of pollutants in the leachate of #1-5 tailing sand, the amount of tailing sand added in each layer had a great impact on the pollutants in the tailing sand immobilized by the SRB. As a whole, the ability of SRB to immobilize the pollutants in the tailings decreased with the increase in the amount of tailings added in each layer. When 1 cm of tailings (#1) was added to each layer, the immobilization percentage of ${\text{SO}}_{4}^{2-}$, Fe(III), Mn(II), Pb(II), Zn(II), Cu(II) and total Cr in the leachate after SRB treatment of tailings were 95.44%, 100%, 90.88%, 100%, 96.20%, 86.23%, and 93.34%, respectively.

**Figure 6:**
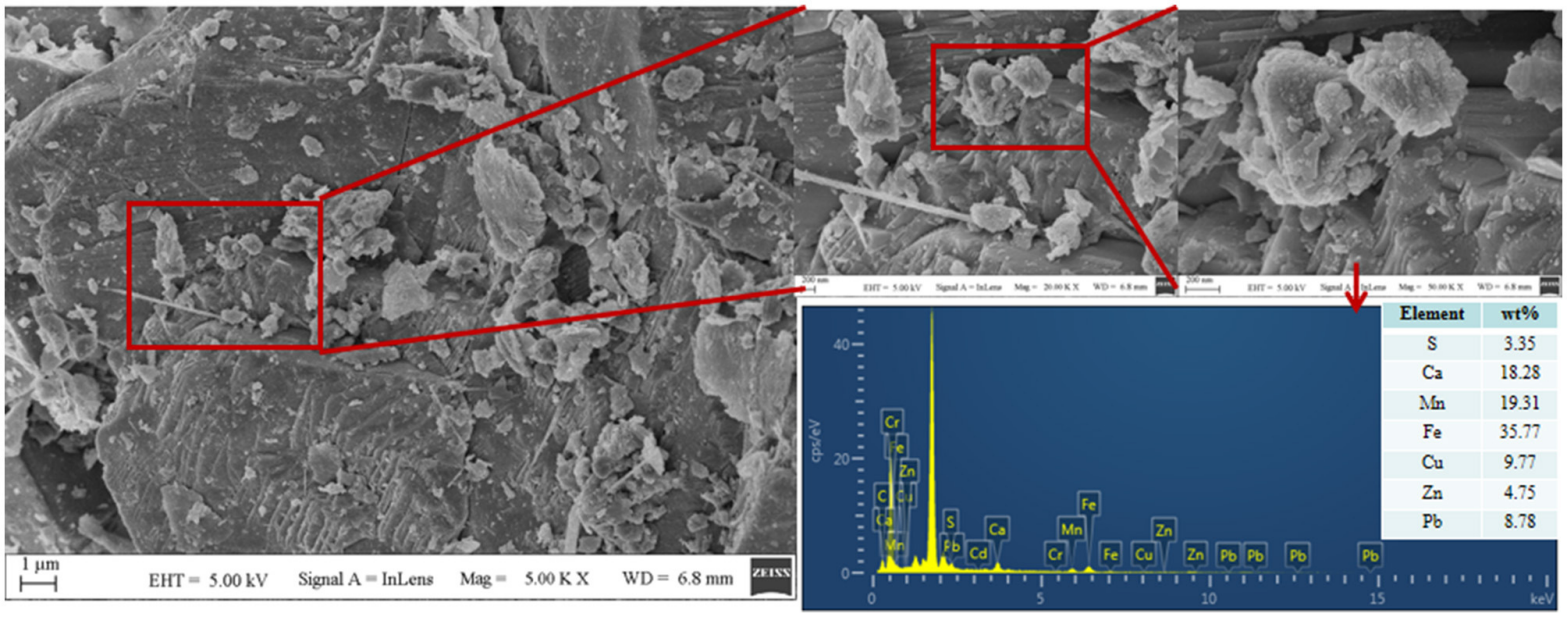
SEM-EDS diagram of #1 tailing sand.

**Figure 7:**
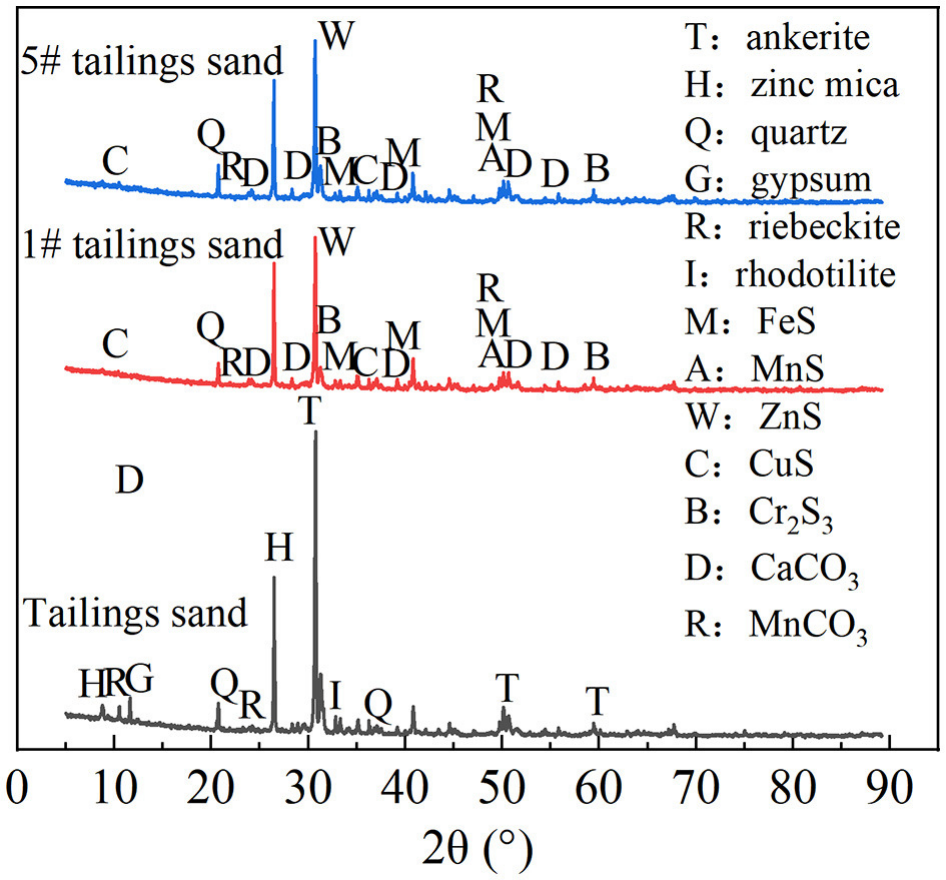
XRD diagram of tailings.

**Figure 8:**
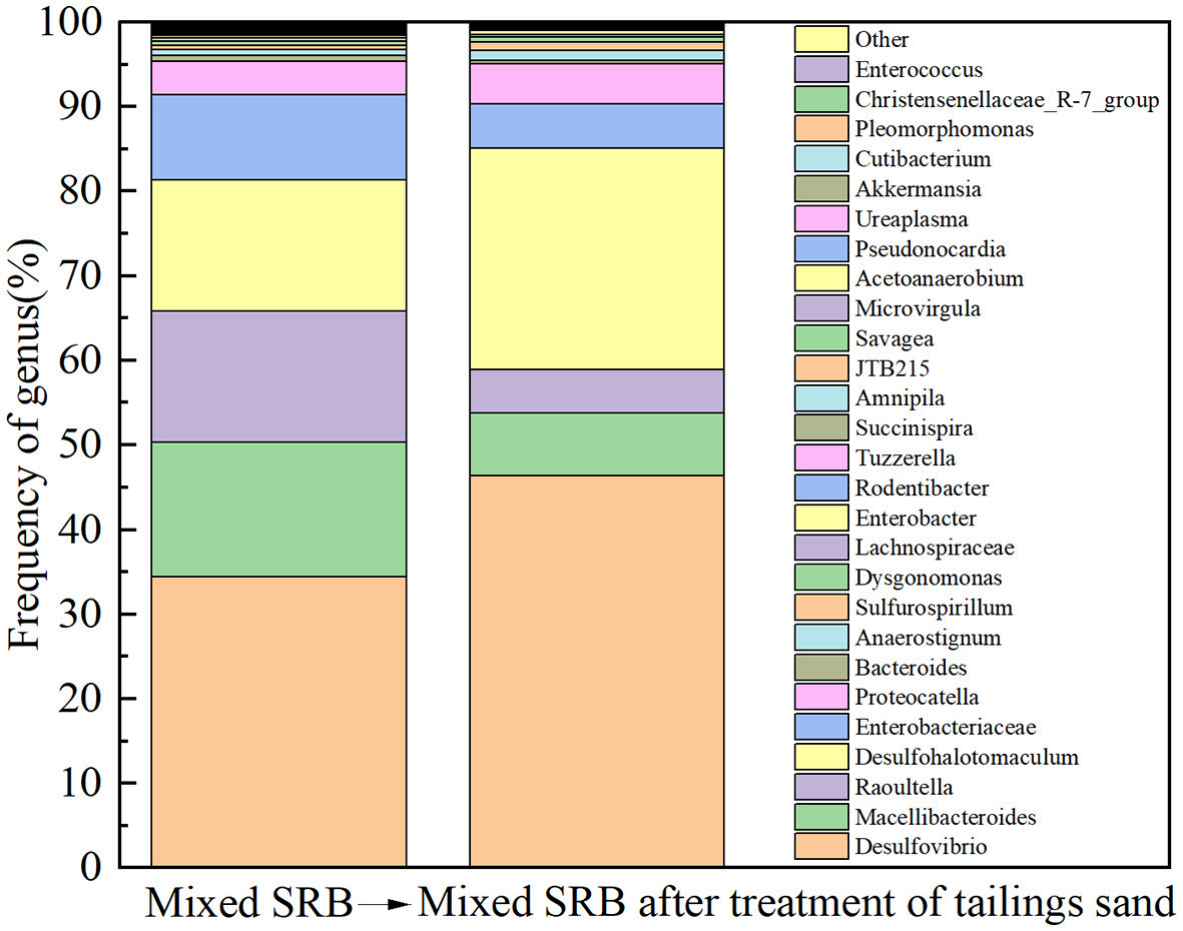
Analysis of microbial community structure of mixed SRB (before and after tailings treatment) at the genus level.

Tailing sand from #1 was taken for SEM-EDS, XRD, and XRF detection. The results are shown in Figures 6, 7, Table 1. The community structure analysis of the mixed SRB before and after the tailings treatment is shown in Figure 8.

**Table 1:** Main chemical composition of tailings before and after solidification (%).

**Component**	**Na_2_O**	**MgO**	**Al_2_O_3_**	**SiO_2_**	**SO_3_**	**K_2_O**	**CaO**	**Cr_2_O_3_**	**MnO**	**Fe_2_O_3_**	**CuO**	**ZnO**	**PbO**
Tailing sands	0.13	10.03	2.92	24.48	7.25	0.77	18.43	0.12	19.23	15.22	0.23	0.40	0.38
1# tailing sands	0.01	9.44	3.07	23.87	6.50	0.76	16.69	0.02	19.45	17.18	0.06	0.41	0.39

It can be seen from Figure 6 that a large number of small particles appear on the surface of #1 tailing sand particles. An EDS analysis of these small particles shows that the contents of S, Ca, Cr, Mn, Fe, Cu, Zn, Cd, and Pb in the particles are 3.35%, 18.28%, 0%, 19.31%, 35.77%, 9.77%, 4.75%, 0%, and 8.78%, respectively. It shows that after SRB treatment, the soluble ionic substances in the tailings were converted into precipitates and attached to the surface of the tailings. These precipitates formed by SRB mainly contained S, Ca, Mn, Fe, Cu, Zn, Pb, and other elements. It can be seen from Figure 7 that the tailing sand is mainly composed of quartz, ankerite, zinc-mica, sodium amphibole, gypsum, rhodochrosite, and other minerals. Among them, iron dolomite, zinc mica, sodium amphibole, gypsum, rhodochrosite, and other minerals easily dissolve and produce pollutants such as sulfate and heavy metal ions. FeS, MnS, ZnS, CuS, Cr_2_S_3_, CaCO_3_, MnCO_3_, and other new substances appeared in #1 and #5 tailing sand. It shows that SRB can metabolize sulfate to produce S^2−^ and carbonate and other metabolites when processing tailings. These metabolites combine with heavy metal ions released from tailings to form precipitated particles, which then get attached to the surface of the tailings. It can be seen from the XRF test results in Table 1 that the main chemical components in the tailings change before and after the SRB treatment. The content of Na_2_O, MgO, SO_3_, Cr_2_O_3_, and CuO in the tailings treated by SRB is significantly reduced, which is related to the dissolution of minerals in the solidification process. In particular, the reduction of S content is related to the release of large amounts of sulfate from tailings leaching. The content of MnO, Fe_2_O_3_, ZnO, and PbO in #1 tailing sand formed after SRB treatment is significantly increased, which is related to the deposition of Mn, Fe, Zn, and Pb in the tailing sand after plasma dissolution and reaction with SRB's metabolic products S^2−^, ${\text{CO}}_{3}^{2-}$, and other reactions to adhere to the surface of tailing sand.

To sum up, the iron dolomite, zinc mica, sodium amphibole, gypsum, and red silicon calcium manganese ore in the tailing sand are easily dissolvable and produce pollutants such as sulfate and heavy metal ions. SRB can metabolize sulfate to produce S^2−^ and carbonate and other metabolites when processing tailings. These metabolites combine with heavy metal ions released from the tailings to form FeS, MnS, ZnS, CuS, PbS, Cr_2_S_3_, CaCO_3_, MnCO_3_, and other precipitated particles, which are attached to the surface of tailings.

According to Figure 8, prior to treating the tailings, the mixed SRB community at the genus level is primarily composed of *Desulfovibrio, Macellibacteroides, Raoultella, Desulfohalotomaculum, Enterobacteriaceae, Proteocatella, Bacteroides, Anaerostignum, Sulfurospirillum, Dysgonomonas, Lachnospiraceae*, and *Enterobacter*, with relative abundances of 34.38%, 15.92%, 15.54%, 15.51%, 10.02%, 4.02%, 0.67%, 0.66%, 0.53%, 0.44%, 0.4%, and 0.3%, respectively. After treating the tailings, the mixed SRB community at the genus level is primarily composed of *Desulfovibrio, Macellibacteroides, Raoultella, Desulfohalotomaculum, Enterobacteriaceae, Proteocatella, Bacteroides, Anaerostignum*, and *Sulfurospirillum*, with relative abundances of 46.42%, 7.34%, 5.11%, 26.18%, 5.31%, 4.72%, 0.42%, 1.15%, and 0.98%, respectively. Comparing the relative abundances of the organisms before and after treating the tailings, it can be observed that *Desulfovibrio, Desulfohalotomaculum, Proteocatella, Anaerostignum, Sulfurospirillum, Enterobacter*, and *Dysgonomonas* increased by 12.04%, 10.67%, 0.70%, 0.49%, 0.45%, 0.15%, and 0.13%, respectively. *Desulfovibrio* and *Desulfohalotomaculum*, belonging to SRB, and *Sulfurospirillum*, associated with sulfur metabolism, are the primary bacteria involved in the sulfate reduction of lead-zinc tailings. The increase in their relative abundances indicates that the high concentration of sulfate released from lead-zinc tailings promotes the growth and proliferation of sulfur-metabolizing bacteria. Additionally, this suggests that the concentration of heavy metals released from the tailings is not sufficient to suppress the growth of sulfur-metabolizing bacteria. Considering the metabolic products after treating the tailings, it can be inferred that FeS, MnS, ZnS, CuS, PbS, Cr_2_S_3_, CaCO_3_, and MnCO_3_ formed after the mixed SRB treatment of tailings are primarily metabolized by *Desulfovibrio* and *Desulfohalotomaculum*.

### 3.4 Discussion

In this study, SRB had a strong ability to remove Zn(II) and Pb(II) in the solution state. The maximum value of pH, removal percentage of ${\text{SO}}_{4}^{2-}$, and percentage removal of Zn(II) were 6.76, 75.72, and 76.69%, respectively, and the minimum value of ORP was−344 mV when Zn(II) solution of 40 mg/L was treated with SRB. The pH value and Pb(II) removal percent were 6.62 and 100%, and the minimum value of ORP was−341 mV. It has been reported that the ORP value is an important indicator in SRB reaction activity; when the ORP value was below−100 mV, it was favorable to maintain the SRB activity, and when the ORP value was higher than−100 mV, it would inhibit SRB activity (Xu and Chen, 2020). The ORP values of SRB in Zn(II) and Pb(II) solutions were much lower than−100 mV, indicating that SRB could maintain higher activity in Zn(II) and Pb(II) solutions.

The main mechanism of Zn(II) and Pb(II) removal by SRB is that SRB metabolizes organics and ${\text{SO}}_{4}^{2-}$ to produce S^2−^ and ${\text{CO}}_{3}^{2-}$. Meanwhile, S^2−^ together with ${\text{CO}}_{3}^{2-}$ reacts with the Zn(II) and Pb(II) in the solution to form precipitation to remove Zn(II) and Pb(II). Besides, it produces large amounts of extracellular polymeric substances (EPS) when SRB activity is high. It has been reported that EPS had the ability to adsorb heavy metal cations, which subsequently combined with S^2−^, and it was a key step in the microbial synthesis of metal sulfide crystals (Su et al., 2022). It has also been reported that a concentration of 45 mg/L of Zn(II) in AMD was a high concentration in wastewater (Gandy et al., 2023). As for the biological treatment of high concentrations of Zn(II)-containing AMD, Gandy et al. (2023) found that the addition of a liquid carbon source (propionic acid) could help to increase the SRB activity, resulting in the removal percent of Zn(II) reaching 99% after treating for 193 days. The removal of Zn(II) by SRB in this study (76.69%) was relatively low compared to the study above, but the removal period in this study was only 8 days, which was much shorter than the study period above.

The long cycle of SRB growth consumed large amounts of organics, increasing the cost of SRB treating Zn(II). Nielsen et al. (2018) established a bioreactor with waste rock and SRB using polyethylene drums on the floor of the Silver King mine. The initial concentrations of Zn(II) and Cd(II) flowing into the reactor were 640.4 μg/L and 10.5 μg/L, with a hydraulic retention time (HRT) of 2 weeks, and the removal rates of Zn(II) and Cd(II) reached 80.6% and 90.5% after treating. Compared with the studies above, the removal rate of Zn(II) by SRB in this study was relatively high, and the removal period was relatively short, which greatly saved the cost of SRB treating heavy metal-containing wastewater.

Miao et al. (2018) evaluated the ability of a natural strain of SRB called *Desulfuromonas alkenivorans S*-7 to remove acidic water (pH=6.0) containing different Pb(II) concentrations (1.0–11.6 mg/L) and showed that strain *S*-7 could remove 90.0% of Pb(II) after 7 days of cultivation. Gopi Kiran et al. (2018) used sodium alginate to immobilize SRB for batch heavy metal removal experiments and found that insoluble metal sulfides were formed outside the surface of sodium alginate immobilized SRB particles, and the removal percentage in 25 mg/L Zn(II) and 25 mg/L Pb(II) was higher than 95%. Compared to the studies above, the removal percentage of Pb(II) by SRB in this study was relatively high at 100% for 50 mg/L Pb(II).

Zhang et al. (2019) protected SRB by wrapping it with a complex of polyvinyl alcohol and sodium alginate and used this SRB to treat the solution with Pb(II). They found that the adsorption of Pb(II) increased with the addition of initial Pb(II) concentration. When the 50 mg/L of Pb(II) in the reaction system was at 35°C and pH=8.0, the removal efficiency reached the highest, with a maximum adsorption capacity of 707.3 mg/g. Their study showed that the complex containing polyvinyl alcohol and sodium alginate could protect SRB from slightly acidic and heavy metal ions. On the other hand, in this study, the SRB without any protection from other materials could still reach a similar removal capacity to 50 mg/L Pb(II). It indicated that the SRB enriched from this study had a strong tolerance to Pb(II). Above all, the SRB used in this study was more tolerant to Zn(II) and Pb(II) and could reach relatively high efficiency in removing Zn(II) and Pb(II) in a short time cycle. It was more suitable for removing tailing sand loaded with heavy metals.

Achal et al. (2013) found that the compressive strength of the chrome slag brick after being immobilized with *Bacillus sp. CS*8 was approximately at 0.36 Mpa, which prevented Cr(VI) contamination in the surrounding environment. Hataf and Baharifard (2019) treated landfill soil with *Bacillus sphericalus*. The results showed that *Bacillus sphaericus* could successfully reduce the permeability of landfill soils and form an effective protective barrier. However, solid waste such as chromium slag and landfill soil can release large amounts of contaminant ions. The studies above analyzed the stabilization effect of solid waste by bacteria only from the perspective of mechanical analysis, such as compressive strength and permeability, and ignored the diffusion of polluting ions. In this study, the fixed percentage of ${\text{SO}}_{4}^{2-}$, Fe(III), Mn(II), Pb(II), Zn(II), Cu(II), and total Cr in tailing sand leachate by SRB reached 95.44%, 100%, 90.88%, 100%, 96.20%, 86.23%, and 93.34%, respectively.

Following SRB treatment of tailings, although the tailings could solidify from loose granular particles into a cohesive mass, the UCS of the specimens remained below 0.2 MPa. Research has indicated that after using *Bacillus subtilis* to cement and solidify artificially simulated lead-contaminated soil, the UCS of the soil increased from 0.065 MPa to 0.525–0.539 MPa (Hadiz et al., 2022). Achal et al. (2013) utilized *Bacillus sp*. CS8 to cement and solidify chromium-contaminated soil, resulting in a UCS increase of 0.36 MPa. Some scholars have also used *T. tumescens* to cement and solidify sand soil, resulting in a UCS of 0.67 MPa (Meng et al., 2016). Preliminary experimental studies have shown that a mixed culture of *Sporosarcina pasteurii* can cement and solidify lead-zinc tailings, with UCS reaching 0.9 MPa after treatment (Dong, 2023a,b). Compared with these, in this study, the UCS after SRB treatment of tailings was significantly lower than that achieved with Bacillus. This indicates that although SRB can undergo microbially induced calcium carbonate precipitation (MICP), the cementation and solidification effect of MICP is not as ideal as that of Bacillus.

The mineralized products formed after SRB treatment of tailings are primarily sulfides, with a small amount of CaCO_3_. The main factors determining the effectiveness of MICP cementation and solidification are the production and adhesive properties of biogenic CaCO_3_. The CaCO_3_ formed after SRB treatment of tailing sand exhibits a binding effect capable of cementing the tailing sand. However, due to the limited amount of CaCO_3_ generated, the lead-zinc tailing sand treated with SRB could only transition from a loose granular state to a solidified mass, resulting in relatively low UCS values. Compared to untreated lead-zinc tailings, the heavy metal ions in the tailings were effectively immobilized after the SRB treatment. Additionally, although the amount of biogenic CaCO_3_ produced through MICP by SRB was relatively low, it still contributed to the cementation and solidification of some tailings particles. This helps alleviate the potential for landslides and debris flows during tailings deposition.

Zhu et al. (2016) found that the urease-producing bacteria *Bacillus cereus NS*4 had the ability to reduce the soluble changeable part of Ni to 38 mg/kg. Govarthanan et al. (2013) used *Bacillus sp. KK*1 to stabilize the Pb^2+^ in soil and found that the content of PbCO_3_ in the soil after treatment increased by 38%. Zhao et al. (2017) used carbonate biomineralizing microorganisms *GZ-*22 enriched from the mining soils. The bacteria had the strongest ability to remove Cd of 10 mg/L at 48 h, in the way of turning Cd into CdCO_3_, and the removal percentage was 60.72%. The studies above showed that the bacteria had a good fixation effect on Ni and Pb in the soil or solution. However, most studies focused on a particular heavy metal ion. In this study, the tailing sand was the sample soil enriched from the mine site, which contained a more complicated composition of heavy metals. In the study of solidifying soil, many kinds of heavy metal pollutants may exist in the soil at the same site. Therefore, many researchers have attempted to precipitate mixtures of heavy metal ions using multiple types of bacteria or in the composite method of combining bacteria with other materials (Chen and Tian, 2021). Hu et al. (2021) found that with a continuous biofiltration treatment using biochar as the medium, nearly 100% removal percentage of As, Cd, Mn, and Ni can be obtained in the conditions of co-treatment of shale oil and gas-produced water (PW) with synthetic domestic wastewater (SDW) (PW:SDW=1:1) and urea concentration of 4 g/L. Compared to the combination of microorganisms, biochar, PW, and SDW in the above studies, this study achieved higher fixation efficiency for all heavy metals using only the combination of microorganisms and tailings, which was simpler and less costly.

In this study, SRB not only immobilized heavy metal ions in the tailing sand but also raised the pH of the tailing sand leachate from 6.21–6.31 to 7.41–7.81. It indicated that the metabolites of SRB could raise the pH and that a higher pH value was conducive to the immobilization of metal ions. A few studies also obtained the same conclusions (Nguyen et al., 2020; Nogueira et al., 2021; Chai et al., 2023). Nguyen et al. (2020) isolated an acid-tolerant sulfate-reducing bacterium, strain *S*4, which could reduce sulfate in a low pH environment and was resistant to very high concentrations of heavy metals (Fe of 3,000 mg/L, Zn of 100 mg/L, and Cu of 100 mg/L). Chai et al. (2023) found that SRB had the ability to raise the pH value when cultivating SRB using organic waste. The removal percentage of sulfate in the solution could reach 95.6%, and the highest removal percentage of Al and Mn were 99.0% and 96%, respectively. Nogueira et al. (2021) treated artificial synthesis of AMD using SRB as the fillers of underflow structured bed bioreactors and found that SRB could increase the pH from moderately acidic to 6.7–7.5; the removal percentage of sulfate was 55%-91%, and the removal percentage of Fe, Zn, Cu, and Mn were 70%, 80%, 73%, and 60%, respectively. Most of these studies aimed to raise the pH when removing sulfate and heavy metal ions from the solution using SRB. Compared to the studies above, this study showed that SRB could raise the pH value not only in solutions but also in solid soil leachate and effectively reduce the release of contaminants from tailing soils.

Liu et al. (2021) found that soluble Pb, Zn, and Cd were reduced by 33.3–85.9%, 21.4–66.0%, and 13.6–29.9%, respectively, with Pb, Zn, and Cd mostly forming carbonate precipitates after bioremediation while using the urease-producing bacteria *sporosarcina pasteurii* to remedy the contaminated soils polluted by Pb, Zn, and Cd; the pH value of the soil was 7.41. Chen and Achal (2019) proved that the ureolytic bacteria in the soil mainly turned Cu(II) into CuCO_3_ to immobilize Cu(II), and the soluble, exchangeable part of Cu decreased from 45.54 mg/kg to 1.55 mg/kg, with an initial soil pH value of 7.11. Peng et al. (2020) isolated a type of ureolytic bacteria *Enterobacter sp*. which had a maximum immobilization percentage of 56.10% for Cd(II) in the soil, and the pH of the leachate was at ~8. The mechanism of removing Cd was mainly through the absorption of Cd(II) by biominerals, including calcite and sphalerite. Similarly, Xu et al. (2019) found that *S. pasteurii* (CGMCC1.3687) could effectively reduce the leachability of heavy metals in municipal solid waste incineration fly ash with a pH value of 10.8. The leaching concentrations of Zn, Cu, Pb, Cr, and Cd after treatment were 0.048, 1.16, 0.005, 0.065, and 0.001 mg/L, respectively. And the unconfined compressive strength could reach approximately 0.16 MPa. Above all, bacteria, such as *sporosarcina pasteurii*, had a good immobilization effect on heavy metals in soil; the mechanism of immobilizing heavy metals by these bacteria is mostly related to the formation of carbonate precipitates. Meanwhile, the pH background of the soil in the studies above mostly appeared as neutral and alkaline (ranging between 7.11 and 10.8). In this study, the pH of the tailing sand leachate was 6.21–6.31, which was weakly acidic. Compared to the alkaline environment, weakly acidic environments could intensify the release of heavy metal contaminants from tailing sand and were less conducive to the remediation of heavy metals by bacteria.

High concentrations of metal cations may even have toxic or inhibitory effects on bacterial communities (Ayangbenro et al., 2018). Meanwhile, the pH value may have an effect on the form of carbonate precipitation. Usually, at the same pH value conditions, from the solubility product, we know that sulfide precipitation of heavy metals was more stable than hydroxide precipitation or carbonate precipitation (Kumar et al., 2021; Su et al., 2022). Therefore, compared to carbonate-producing bacteria such as *sporosarcina pasteurii* in this study, the heavy metal ions in the tailing sand immobilized by SRB were more stable.

Yan et al. (2020) found that mixed SRB, including *Desulfovibrio, Desulfomicrobium*, and *Desulfococcus*, were effective in removing sulfate from AMD. Hái and Hă°ng (2016) enriched mixed SRB from wastewater generated in aquaculture processing. This mixed SRB mainly consisted of *Desulfovibrio, Desulfomicrobium*, and *Desulfobulbus spp*. These mixed SRB exhibited a fixation rate of 85–88% for Fe^2+^ in AMD (with an initial Fe^2+^ concentration of 200 mg/L) (Hái and Hă°ng, 2016). Walters et al. (2013) conducted six field-scale AMD remediation column experiments at the Tab-Simco site. The results indicated that SRBs such as *Desulfotomaculum* and *Desulfococcus* in the microbial community exhibited strong remediation capabilities for sulfate and heavy metals in AMD. Dev et al. (2021) reported that SRB, including *Desulfosporosinus* and *Desulfotomaculum*, play a significant role in the effective biological treatment of AMD in cold regions.

In summary, *Desulfovibrio, Desulfomicrobium, Desulfococcus, Desulfobulbus spp*., *Desulfotomaculum, Desulfosporosinus*, and similar SRB species all belong to sulfate-reducing bacteria and are capable of driving sulfate reduction, thus facilitating the bioremediation of AMD. Considering the findings from the aforementioned studies and the biodiversity of mixed SRB in this study, it is evident that *Desulfovibrio* and *Desulfohalotomaculum* play a predominant role in treating tailings. These bacteria metabolize the sulfate and heavy metals released from the tailings, forming precipitate products such as FeS, MnS, ZnS, CuS, PbS, Cr_2_S_3_, CaCO_3_, and MnCO_3_. This process mitigates pollution by tailings while cementing and solidifying tailings particles, thereby improving the mechanical properties of the tailings. Above all, this study demonstrated that SRB is more suitable for environmental remediation of tailing sand in the mining environment, and it is a technique for *in situ* remediation of multiple contaminants in tailing sand.

## 4 Conclusion

To address heavy metal pollution in tailings and AMD, soil-enriched SRB was employed using microbial treatment technology. By analyzing the treatment effects of SRB on simulated solutions containing different concentrations of Pb(II) and Zn(II), the tolerance of SRB to Pb(II) and Zn(II) concentrations was investigated. The pollutants released from lead-zinc tailings are more complex than those from AMD. By analyzing the fixation effect of SRB on heavy metal pollutants in tailings, the generation of heavy metal-containing wastewater can be reduced from the source, thereby lowering the cost of environmental management in mining areas. Combined with UCS, the cementing strength of SRB on tailings particles was analyzed from a mechanical perspective. Although the UCS strength is relatively low, in the future, the strength of SRB cemented tailings can be enhanced from the perspectives of fiber reinforcement and reinforced solidification, providing technical references for bio-cementation reinforcement technology of tailings particles in mining areas and mitigating the impact of geological disasters such as tailing slides and debris flows. Additionally, combining SEM-EDS, XRF, and XRD with other detection methods revealed the mechanism of SRB fixation of pollutants in the tailings. This method provides technical references for the effective and sustainable remediation of AMD and tailings by SRB, promoting the implementation of green mining construction.

(1) SRB had a strong removal capacity for Zn(II) in the concentration range of 0-40 mg/L. If the initial concentration of Zn(II) is increased to 60-100 mg/L, then the growth of SRB would be inhibited. The tolerance concentration of SRB to Zn(II) is 40 mg/L. At this concentration, the maximum values of OD_600_, pH value, and immobilization percentages of ${\text{SO}}_{4}^{2-}$ and Zn(II) were 0.92, 6.76, 75.72%, and 76.69% respectively, and the minimum values of ORP and Ec were−344 mV and 2.77 mS/cm respectively.

(2) SRB had a very strong ability to remove Pb(II). When the initial Pb(II) concentration was 10 mg/L, 20 mg/L, 30 mg/L, 40 mg/L, and 50 mg/L, respectively, the removal percentages of ${\text{SO}}_{4}^{2-}$ were 77.95%, 76.04%, 76.33%, 69.81%, and 66.45% after adding SRB for 8 days. The final removal percentage of Pb(II) reached 100%.

(3) SRB treatment can effectively fix ${\text{SO}}_{4}^{2-}$, Fe(III), Mn(II), Pb(II), Zn(II), Cu(II), total Cr, and other pollutants leached from tailings, and reduce environmental pollution of tailings to water and soil around the mining area. At the same time, with an increase in the amount of tailings added to each layer, the ability of SRB to treat the pollutants in the tailings decreased. After SRB treatment, although the tailings can be solidified from loose granular particles into a cohesive mass, the UCS of the specimens remains below 0.2 MPa. The enhancement of strength in SRB-treated tailings awaits further research in directions such as fiber reinforcement and reinforced curing. When 1 cm of tailing sand was added to each layer (#1 tailing sand), SRB had the best effect on treating tailing sand. The removal percentages of ${\text{SO}}_{4}^{2-}$, Fe(III), Mn(II), Pb(II), Zn(II), Cu(II), and total Cr in the leachate of #1 tailing sand after SRB treatment were 95.44%, 100%, 90.88%, 100%, 96.20%, 86.23%, and 93.34%, respectively.

(4) The iron dolomite, zinc mica, sodium amphibole, gypsum, and red silicon calcium manganese ore in the tailing sand were easy to dissolve and produce pollutants such as sulfate and heavy metal ions. *Desulfovibrio* and *Desulfohalotomaculum* play a predominant role in treating tailings within the mixed SRB. Mixed SRB can metabolize sulfate to produce S^2−^ and carbonate and other metabolites when metabolizing tailings. These metabolites combine with heavy metal ions released from tailings to form FeS, MnS, ZnS, CuS, PbS, Cr_2_S_3_, CaCO_3_, MnCO_3_, and other precipitated particles, which were attached to the surface of tailings.

## Data availability statement

The original contributions presented in the study are included in the article/supplementary material, further inquiries can be directed to the corresponding author.

## Author contributions

YD: Writing – original draft. ZG: Writing – review & editing. JD: Writing – review & editing. DW: Writing – review & editing. ZY: Writing – review & editing. XG: Writing – review & editing. XZ: Writing – review & editing.
